# Transcriptomic Profiling of Mouse Brain During Acute and Chronic Infections by *Toxoplasma gondii* Oocysts

**DOI:** 10.3389/fmicb.2020.570903

**Published:** 2020-10-19

**Authors:** Rui-Si Hu, Jun-Jun He, Hany M. Elsheikha, Yang Zou, Muhammad Ehsan, Qiao-Ni Ma, Xing-Quan Zhu, Wei Cong

**Affiliations:** ^1^State Key Laboratory of Veterinary Etiological Biology, Key Laboratory of Veterinary Parasitology of Gansu Province, Lanzhou Veterinary Research Institute, Chinese Academy of Agricultural Sciences, Lanzhou, China; ^2^College of Animal Science and Technology, Jilin Agricultural University, Changchun, China; ^3^Faculty of Medicine and Health Sciences, School of Veterinary Medicine and Science, University of Nottingham, Loughborough, United Kingdom; ^4^College of Veterinary Medicine, Shanxi Agricultural University, Taigu, China; ^5^Marine College, Shandong University, Weihai, China

**Keywords:** *Toxoplasma gondii*, oocysts, RNA-seq, transcriptome, cerebral toxoplasmosis, host-parasite interaction

## Abstract

Infection by the protozoan *Toxoplasma gondii* can have a devastating impact on the structure and function of the brain of the infected individuals, particularly immunocompromised patients. A systems biology view of the brain transcriptome can identify key molecular targets and pathways that mediate the neuropathogenesis of cerebral toxoplasmosis. Here, we performed transcriptomic analysis of the brain of mice infected by *T. gondii* Pru strain oocysts at 11 and 33 days post-infection (dpi) compared to uninfected (control) mice using RNA sequencing (RNA-seq). *T. gondii* altered the expression of 936 and 2,081 transcripts at 11 and 33 dpi, respectively, and most of these were upregulated in the infected brains. Gene Ontology (GO) enrichment and pathway analysis showed that immune response, such as interferon-gamma (IFN-γ) responsive genes were strongly affected at 11dpi. Likewise, differentially expressed transcripts (DETs) related to T cell activation, cytokine production and immune cell proliferation were significantly altered at 33 dpi. Host-parasite interactome analysis showed that some DETs were involved in immune signaling, metabolism, biosynthesis-related processes and interspecies interaction. These findings should increase knowledge of the mouse brain transcriptome and the changes in transcriptional regulation and downstream signaling pathways during acute and chronic *T. gondii* infections.

## Introduction

*Toxoplasma gondii* is a widely prevalent intracellular apicomplexan protozoan parasite which chronically infects approximately one-third of the world’s population (Tenter et al., 2000). Although infection by *T. gondii* does not normally cause clinical illness in humans, individuals whose immune system is compromised can develop serious health problems. *T. gondii* has the ability to infect all tissue types in the affected host, however, this parasite has preference for the brain and muscles, where it remains for a long time in these tissues as dormant cysts. Chronic *T. gondii* infection can lead to neurological disorders, such as bipolar disease and schizophrenia (Elsheikha et al., 2016; Elsheikha and Zhu, 2016; Shiojiri et al., 2019). A correlation between dysregulated immune-inflammation and brain dysfunctions during *T. gondii* infection has been discussed previously (Parlog et al., 2015; Elsheikha and Zhu, 2016; Wohlfert et al., 2017). Additionally, the dormant *T. gondii* cysts can reactivate and cause severe encephalitis, leading neurological manifestations and even fatal consequences (Wohlfert et al., 2017). *T. gondii* infections represent one of the major health challenges that compromise the life of people with HIV and remain a prominent cause of morbidity and mortalities in immunocompromised patients in general (Mayor et al., 2011). One study reported that 65% of HIV patients died within the first year of diagnosis with reactivated latent toxoplasmosis (Mayor et al., 2011).

How *T. gondii* reaches and colonizes the brain and the molecular mechanisms that underpin these processes continue to challenge scientists. The neuro-pathogenesis of *T. gondii* infection is driven by a complicated network of molecular processes and pathways (Ngo et al., 2017). Data supporting this view are emerging from various “-omics”-based profiling of the brains of mice infected by *T. gondii* (Jia et al., 2013; Tanaka et al., 2013; Pittman et al., 2014; Zhou et al., 2015; Hu et al., 2018; Garfoot et al., 2019a; Ma et al., 2019). The magnitude of this challenge has increased the interest in understanding how *T. gondii* infection affects murine host at the transcriptional level in various tissues other than the brain, including the liver (He et al., 2016a), spleen (He et al., 2016b,c), peripheral lymphocytes (Jia et al., 2013), and the uterus (Zhou et al., 2020). Increased understanding of the brain transcriptomic signature associated with *T. gondii* infection can provide new testable hypotheses that may ultimately facilitate the development of new treatment interventions to control cerebral toxoplasmosis. However, the effect of infection by *T. gondii* oocysts on the brain transcriptome remains obscure, although consumption of oocysts with food or water contaminated with cat feces represents a major route of infection of the intermediate hosts (Dubey, 2009; Blader et al., 2015).

In the present study, the transcriptomic response of mouse brain to experimental *T. gondii* infection by oocysts of type II Pru strain was examined using RNA sequencing (RNA-seq). Our computational analysis revealed significant transcriptomic changes in the infected and uninfected (control) mice at 11 and 33 days post-infection (dpi). We identified 936 differently expressed transcripts (DETs) during acute (11 dpi) infection and 2,081 DETs during chronic (33 dpi) infection. The transcriptional changes correlated with *T. gondii* load in infected brain tissues. The identified DETs were involved in metabolism, immune-related, biosynthesis-related processes, and interspecies interaction between organisms. Our data not only show how *T. gondii* oocyst infection alters the transcriptome of the brain of mice at early and late stage of infections, but also provide insight for future research efforts into the development of therapeutic interventions to combat *T. gondii* infection of the brain.

## Materials and Methods

### Collection and Preparation of Sporulated Oocysts

One, 10-week-old, specific-pathogen-free (SPF), kitten was fed 100 cysts of Pru strain freshly prepared from brain homogenates obtained from experimentally infected mice. Cat feces were examined microscopically on a daily basis. Oocyst collection was performed as previously described (Zhou et al., 2016), and purification of oocysts was performed using sucrose flotation and cesium chloride gradient as previously described (Staggs et al., 2009). To obtain sporozoites-containing (i.e., infectious) oocysts, the purified oocysts were centrifuged at 360 × *g*, and the oocyst pellet was suspended in 2% sulfuric acid and was aerated on a shaker for 7 days at room temperature. Sporulated oocysts were washed twice with 0.85% normal saline and suspended in 2% sulfuric acid. Finally, oocysts were counted using a hemocytometer and their number was adjusted to 100 oocyst/ml in phosphate buffered saline (PBS), and stored at 4°C until use.

### BALB/c Mice and Parasite Challenge

SPF female BALB/c mice, 7-week-old, were purchased from Lanzhou University Laboratory Animal Centre (Lanzhou, Gansu Province, China). Twelve *T. gondii*-seronegative mice were randomly divided into four groups (3 mice/group): two mouse groups were infected by *T. gondii* oocysts for 11 and 33 days, representing the acutely and chronically infected groups, respectively. Two mouse groups were left uninfected for the same length of times as the matched control groups. The mice were handled following the protocol approved by the Animal Ethics Committee of Lanzhou Veterinary Research Institute, Chinese Academy of Agricultural Sciences. *T. gondii* infection was performed in each mouse by oral gavage with 100 oocysts in 1 ml of PBS. The uninfected (control) mice were sham inoculated with 1 ml of PBS only without any oocysts. Mice were maintained in an Animal Biosafety Level 2 (ABSL-2) containment laboratory, with room temperature (22 ± 0.5°C) under 12/12 h light/dark cycle. All mice were fed commercial diet and had access to purified water *ad libitum*. Following the infection, mice were monitored for the development of clinical signs, such as ruffled hair, and physical inactivity. At 11 dpi and 33 dpi, mice were anesthetized by intraperitoneal injection with 100 μl xylazine (20 mg/ml) and ketamine (1 mg/ml) in PBS, and brain tissues were harvested, quickly washed in PBS, flash frozen in liquid nitrogen, and preserved at −80°C until use.

### Detection of *T. gondii* in the Brain Tissue

The presence of *T. gondii* cysts in infected brain tissues has been confirmed in our previous study of microRNA profiling using a microscope (Hu et al., 2018). Additionally, genomic DNA was extracted from the brain tissues using TIANamp Genomic DNA kit (TianGen^TM^, Beijing, China) and DNA samples were stored at −20°C. PCR amplification of *T. gondii B1* gene was used to further confirm the presence of *T. gondii* in the brain tissue (Cong et al., 2015).

### RNA Extraction and Transcriptome Sequencing (RNA-Seq) Analysis

The RNA of each sample were extracted separately using TRIzol Reagent (Invitrogen, CA, United States) according to the manufacturer’s instructions. The quality of the extracted RNA was checked in order to ensure the high quality of RNA library. The purity (OD_260/280_) of the RNA preparation was examined using a NanoPhotometer^®^ spectrophotometer (IMPLEN, CA, United States). All RNA preparations had an absorbance ratio OD_260/280_ > 1.8. RNA samples were further treated with PQI DNase (Promega, MI, United States) to remove any genomic DNA contaminates. The integrity and concentration of RNA were examined using RNA Nano 6000 Assay Kit (Agilent Technologies, CA, United States) and Qubit^®^ RNA Assay Kit (Life Technologies, CA, United States), respectively. About one microgram of the total RNA per sample (of 3 pooled replicates) was used for the construction of mRNA libraries using NEBNext^®^ Ultra^TM^ RNA Library Prep Kit (Illumina, NEB, United States). Each library was sequenced on an Illumina HiSeq^TM^ 2500 (Illumina, San Diego, CA, United States). Adaptor sequences and low-quality reads were removed using Trimmomatic (v.0.39) (Bolger et al., 2014). Genomic data of *Mus musculus* (Mouse; GRCm38.p6) was downloaded from Ensembl database (Release-100). Transcript abundance was estimated using free-alignment method by Salmon (v.1.2.0) because of its high sensitivity and accuracy for bias-aware quantification (Patro et al., 2017). Expression profile of each transcript was normalized to TPM (transcripts per million) (Dillies et al., 2013). Differential expression analysis was performed by using count-based technology-DEseq2 package (v.3.6.1) (Love et al., 2014). The DETs were identified according to *P*-value < 0.05 and | log_2_ fold-change| ≥ 1.5.

### Verification of DETs by Quantitative Real Time qRT-PCR

Total RNA was treated with 1 μl gDNA Eraser, and 2 μl 5 × gDNA Eraser Buffer at 42°C for 2 min in order to remove residual genomic DNA contaminates. Single cDNA was synthesized using a PrimeScript^TM^ RT Reagent Kit (Takara, Japan). qRT-PCR reaction was carried out using the TB Green^TM^ *Premix Ex Taq*^TM^ II (Takara, Japan). The amplification protocol of qRT-PCR was performed as follows: 95°C for 30 s, 40 cycles of 95°C for 5 s, 60°C for 30 s. Melting curve analysis ranging from 65 to 95°C was performed to ensure the specificity of amplification. The measurements of mRNA were normalized to glyceraldehyde-3-phosphate dehydrogenase (*GAPDH*) and the relative abundances of gene expression were calculated using *C*_T_ method (2^–ΔΔ^*^*C*^*^*T*^ method) (Schmittgen and Livak, 2008). Details of the oligonucleotide primers and results of qRT-PCR analysis used to verify the sequencing data are shown in Supplementary Table 2.

### Bioinformatic Analysis

ClusterProfiler (v.3.6.2) in RStudio^1^ was used for Gene Ontology (GO) and Kyoto Encyclopedia of Genes and Genomes (KEGG) pathway enrichment analysis of the DETs. The clusterProfiler is an ontology-based tool that not only automates the process of biological-term classification and the enrichment analysis of cluster genes, but also enables the analysis and visualization of the modules (Yu et al., 2012). FDR (false discovery rate) was calculated using Benjamini and Hochberg method and adjusted *P*-value < 0.05 was set as the cut-off threshold for the significant enrichment. We used TRRUST (v2) (Han et al., 2018) to analyze the transcriptional regulatory networks of mouse. TRRUST is a manually curated database that provides large number of known (activation and repression) and unknown interactions. In this study, only interactions between the transcription factors (TFs) and their targets belonging to the DETs were selected, and external candidates were excluded. For host-parasite interaction analysis, we retrieved mouse and their relevant *T. gondii* interactions from OTHOHPI database (Cuesta-Astroz et al., 2019). Only host proteins with dysregulation at transcript levels were considered. Interaction network from OTHOHPI was visualized in Cytoscape (v.3.7.0) (Shannon et al., 2003) and functional analysis was performed using Gene Ontology database (Ashburner et al., 2000).

## Results

### Characterization of the Transcriptomic Dataset

**FIGURE 1:**
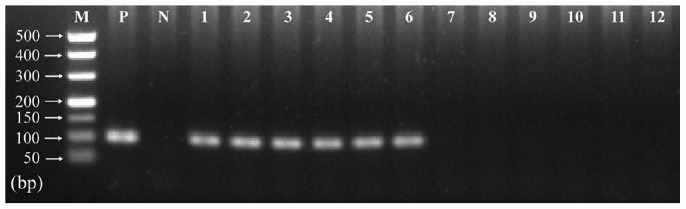
Detection of *T. gondii B1* gene amplified by specific semi-nested PCR in infected and uninfected mouse brain tissues. The amplified band of 98 bp was detected in infected mouse brain (11 and 33 dpi). However, no product was detected in the brain tissues of uninfected mice at the same time points. Lane M: 500 bp DNA molecular ladder; Lane P: positive control; Lane N: negative control; Lanes 1–3: infected brain tissues at 11 dpi; Lanes 4–6: infected brain tissues at 33 dpi; Lanes 7–9: uninfected brain tissues at 11 dpi; Lanes 10–12: uninfected brain tissues at 33 dpi.

**FIGURE 2:**
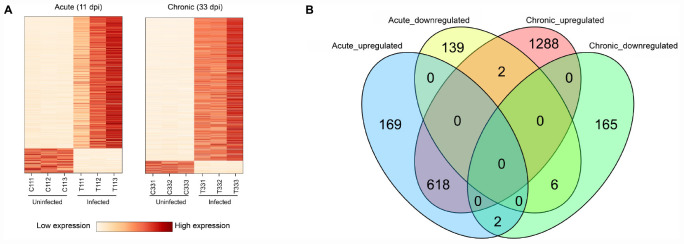
Results of RNA sequencing (RNA-seq) and differential expression analysis of transcripts. **(A)** Heat maps showing the hierarchical clustering of 936 and 2,081 differentially expressed transcripts (DETs) at 11 and 33 dpi, respectively. Each mouse group included three biological replicates. **(B)** Venn diagram showing the overlap of DETs in the brain of acutely- and chronically-infected mice, including upregulated and downregulated transcripts.

The presence of *T. gondii* in the mouse brain at 11 and 33 dpi was confirmed using specific semi-nested PCR primers that target the *B1* gene (Figure 1). The integrity of each RNA template used in the study was >8.0. The high quality RNAs were used to construct mRNA libraries from infected and uninfected brains of mice. In this study, 94% of the reads showed good quality values >Q20, 90% of clean reads was up to Q30, and in each sample >60 million clean reads were generated (Table 1). The results showed clear separation between infected and control transcriptomes and low variations between the biological replicates for all DETs (adjusted *P-*value < 0.05; | log_2_FC| > 1.5) in *T. gondii*-infected and uninfected (control) brains (Figure 2A). In total, 936 DETs (789 upregulated and 147 downregulated) were detected in the brain of acutely infected mice, and 2,081 DETs (1,908 upregulated and 173 downregulated) were detected in the brain of chronically infected mice (Supplementary Table 1). Two pair-wise comparisons of early and late DETs as shown in the Venn diagram, where the upregulated DETs during early and late infections in the brain tissue show that a total of 618 transcripts were shared between acute and chronic infections (Figure 2B). However, in regard to the downregulated DETs, only 6 transcripts were shared (Figure 2B), suggesting a modest commonality in the downregulated DETs during early and late *T. gondii* infections. Ten randomly selected transcripts were confirmed by qRT-PCR, and the results were consistent with those obtained by RNA-seq, supporting the validity of the obtained transcriptomic RNA-seq data (Supplementary Table 2).

**TABLE 1:** Characteristics of the RNA-seq results obtained in the present study.

Mouse groups		Sample code	Raw reads	Clean reads	Clean bases	Error rate (%)	Q20 (%)^a^	Q30 (%)^b^	GC content (%)
11-days post-infection	Infected	T111	68,999,052	65,632,084	9.84G	2.00%	96.08	90.32	49.66
	Infected	T112	70,544,072	67,074,478	10.06G	2.00%	96.03	90.21	49.42
	Infected	T113	69,212,352	65,848,722	9.88G	2.00%	96.09	90.31	49.94
	Control	C111	79,063,632	75,052,192	11.26G	2.00%	96.18	90.54	49.38
	Control	C112	71,692,682	68,026,004	10.2G	2.00%	96.02	99.22	49.74
	Control	C113	70,064,012	66,381,420	9.96G	2.00%	96.06	90.24	49.69
33-day post infection	Infected	T331	68,042,008	66,719,442	10.01G	2.00%	96.71	91.86	48.57
	Infected	T332	71,371,072	69,232,194	10.38G	2.00%	94.96	87.87	49.55
	Infected	T333	67,503,956	64,041,204	9.61G	2.00%	96.06	90.51	50.05
	Control	C331	61,545,672	60,241,390	9.04G	2.00%	96.37	91.90	49.54
	Control	C332	84,972,124	82,414,714	12.36G	2.00%	96.18	90.28	49.87
	Control	C333	70,485,006	69,061,674	10.36G	2.00%	96.81	92.07	50.33

*^*a*^The percentage of bases with a Phred value > 20. ^*b*^The percentage of bases with a Phred value > 30.*

### GO Enrichment and KEGG Analysis

We analyzed the GO functional enrichment and significant pathways associated with upregulated and downregulated transcripts in acutely and chronically infected groups (Supplementary Figure 1). GO terms were classified into three categories: Biological Process (BP), Cellular Component (CC) and Molecular Function (MF).

The upregulated transcripts in the brain tissues of acutely infected mice were significantly enriched in infection- or immune-related processes, such as responses to virus, interferon-mediated host response, antigen processing and presentation, and regulation of innate immune responses. For the upregulated transcripts in the brain tissues of chronically infected mice, most of the transcripts enriched in BP terms were involved in activation of immune cells, such as T-cell activation, lymphocyte proliferation, leukocyte proliferation, and mononuclear cell proliferation. Several significantly enriched pathways in both infection stages exhibited similar patterns, which were involved in disease-related physiological pathways or immune responses, for instance, Epstein-Barr virus infection, viral myocarditis, graft-versus-host disease, Type I diabetes mellitus, phagosome, cell adhesion molecules (CAMs), and antigen processing and presentation pathway.

The downregulated transcripts for the two stages of infection were not enriched in any pathway. However, most significantly enriched CC terms of the downregulated transcripts in early infected mice were involved in cell leading edge, presynaptic and synaptic membrane or their components, as well as cell-cell adherens junctions. The most significantly enriched MF terms of the downregulated transcripts in acutely infected mice were involved in phosphatase binding and extracellular matrix structural constituent. BP terms were not significantly enriched in acutely infected mice. However, during chronic infection, the downregulated transcripts were significantly enriched in BP terms involved in the response to metal and calcium ion. However, CC and MF terms were not significantly enriched during chronic infection.

**FIGURE 3:**
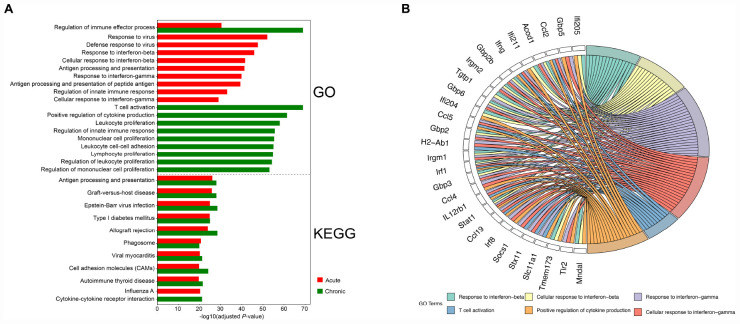
Functional analysis of the differentially expressed transcripts (DETs). **(A)** The top 10 Gene Ontology (GO) terms and KEGG pathways of the DETs detected in the brain of acutely- and chronically-infected mice are shown. **(B)** Circos plot showing the relationship between GO terms and the corresponding genes. A number of genes related to immune response were enriched for biological process such as response to interferon-beta and interferon-gamma, T-cell activation, and positive regulation of cytokine production.

We also performed GO functional enrichment and KEGG analyses of all DETs. According to the adjusted *P*-value, the top 10 significantly enriched GO terms and pathways with respect to reciprocal comparisons of early and late infections showed that genes were mainly involved in biological processed related to immune responses (Figure 3A). There were 6 significantly enriched GO terms that had cross relationships with 28 transcripts (Figure 3B). These included *Acod1*, *Ccl2*, *Ccl4*, *Ccl5*, *Ccl19*, *Gbp2*, *Gbp2b*, *Gbp3*, *Gbp5*, *Gbp6*, *H2-Ab1*, *Ifi204*, *Ifi205*, *Ifi211*, *Ifng*, *Irgm1*, *Irgm2*, *IL12rb1*, *Irf1*, *Irf8*, *Mndal*, *Slc11a1*, *Socs1*, *Stat1*, *Stx11*, *Tgtp1*, *Tmem173*, and *Tlr2*. Notably, all of these transcripts were upregulated in the mouse brain during early and late infections.

### Differentially Expressed Transcription Factors (TFs) and Their Target Genes

**FIGURE 4:**
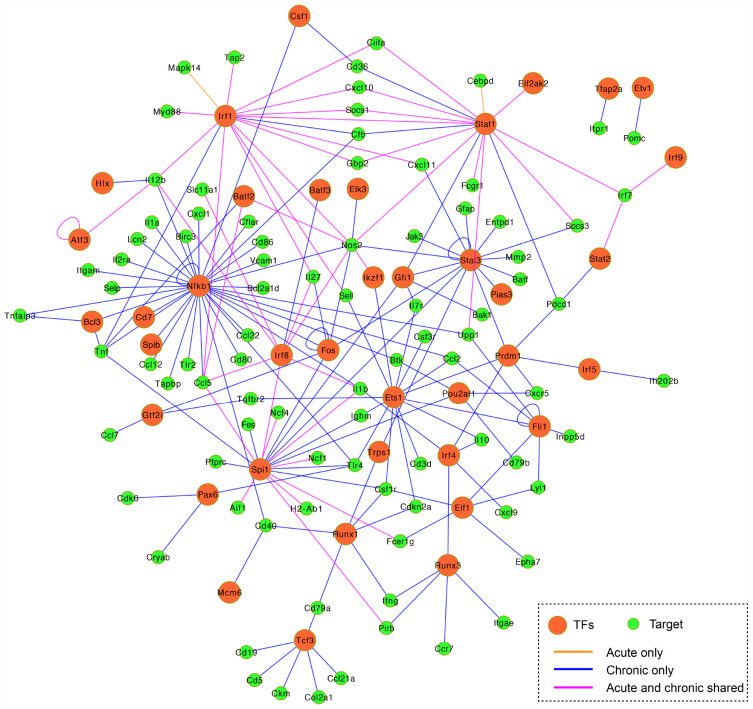
Co-expression regulatory network of the differentially expressed TFs and their target genes. TFs are represented by orange circles and their gene targets are indicated by green circles. The connected lines are shown in pink (shared early and late infections), orange (early infection only), and blue (late infection only). The regulatory mode of the known interactions (i.e., activation and repression) are shown in Supplementary Table 3.

According to TRRUST database, we detected 38 unique differentially expressed TFs (32 upregulated and 6 downregulated) in infected brain tissues (Table 2). TRRUST provides information of known interactions in term of regulatory mode for activation or suppression of TFs interacting with their target genes, each with a variety of evidence types and reference sources. We identified 102 unique DETs targeted by the detected TFs that were involved in 185 unique interactions (Figure 4 and Supplementary Table 3). There were 11 upregulated TFs that were co-activated in both acute and chronic infections (Table 2), which functioned as regulators for shared target genes (interaction lines with purple color); of which, *Irf1* and *Stat1* also can regulate two exclusive target genes in early infection (interaction lines with orange color); and as shown in Figure 4, 35 TFs except *Eif2ak2* and *Irf9* were involved in more interactions together with the corresponding target genes in late stage of infection (interaction lines with blue color).

**TABLE 2:** Description of the differentially expressed transcription factors (TFs) detected in the present study.

Gene symbol	Description^†^	Expression
Atf3*	Activating transcription factor 3	Upregulated
Batf2*	Basic leucine zipper transcription factor, ATF-like 2	Upregulated
Bcl3*	B cell leukemia/lymphoma 3	Upregulated
Eif2ak2*	Eukaryotic translation initiation factor 2-alpha kinase 2	Upregulated
Ikzf1*	IKAROS family zinc finger 1	Upregulated
Irf1*	Interferon regulatory factor 1	Upregulated
Irf8*	Interferon regulatory factor 8	Upregulated
Irf9*	Interferon regulatory factor 9	Upregulated
Spi1*	Spleen focus forming virus (SFFV) proviral integration oncogene	Upregulated
Stat1*	Signal transducer and activator of transcription 1	Upregulated
Stat2*	Signal transducer and activator of transcription 2	Upregulated
Batf3^#^	Basic leucine zipper transcription factor, ATF-like 3	Upregulated
Cd7^#^	CD7 antigen	Upregulated
Csf1^#^	Colony stimulating factor 1 (macrophage)	Upregulated
Elf1^#^	E74-like factor 1	Upregulated
Elk3^#^	ELK3, member of ETS oncogene family	Upregulated
Ets1^#^	E26 avian leukemia oncogene 1, 5′ domain	Upregulated
Fli1^#^	Friend leukemia integration 1	Upregulated
Gfi1^#^	Growth factor independent 1 transcription repressor	Upregulated
Hlx^#^	H2.0-like homeobox	Upregulated
Irf4^#^	Interferon regulatory factor 4	Upregulated
Irf5^#^	Interferon regulatory factor 5	Upregulated
Mcm6^#^	Minichromosome maintenance complex component 6	Upregulated
Nfkb1^#^	Nuclear factor of kappa light polypeptide gene enhancer in B cells 1, p105	Upregulated
Pax6^#^	Paired box 6	Upregulated
Pou2af1^#^	POU domain, class 2, associating factor 1	Upregulated
Prdm1^#^	PR domain containing 1, with ZNF domain	Upregulated
Runx1^#^	Runt related transcription factor 1	Upregulated
Runx3^#^	Runt related transcription factor 3	Upregulated
Spib^#^	Spi-B transcription factor (Spi-1/PU.1 related)	Upregulated
Stat3^#^	Signal transducer and activator of transcription 3	Upregulated
Tcf3^#^	Transcription factor 3	Upregulated
Etv1^#^	Ets variant 1	Downregulated
Fos^#^	FBJ osteosarcoma oncogene	Downregulated
Gtf2i^#^	General transcription factor II I	Downregulated
Pias3^#^	Protein inhibitor of activated STAT 3	Downregulated
Tfap2a^#^	Transcription factor AP-2, alpha	Downregulated
Trps1^#^	Transcriptional repressor GATA binding 1	Downregulated

*Shared differentially expressed TFs between acutely and chronically infected mice. ^#^Differentially expressed TFs in chronically infected mice only. ^†^Functional descriptions of TFs were retrieved from mouse transcriptome dataset (GRCm38.p6; Ensembl database Release-100).

### Mouse Genes Targeted by *T. gondii* Factors

**FIGURE 5:**
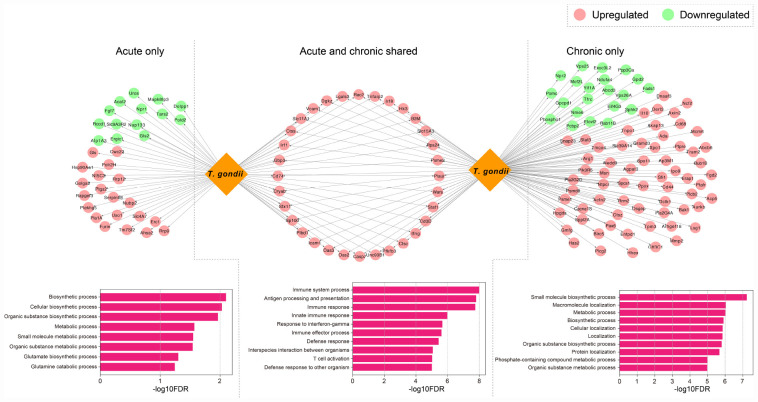
Host-parasite interaction networks and function enrichment analysis of mouse brain genes. DETs were distinguished by acute infection only, shared acute and chronic infections, and chronic infection only. The top 10 biological processes GO terms of the interaction networks are shown.

Based on the identified DETs in infected mouse brain, we detected the host-parasite protein-protein interactions and retrieved 354 computationally predicted interactions (Supplementary Table 4). We found that 159 *T. gondii* proteins related to the secretome and/or the membrane had relevant roles in mouse-*T. gondii* interactome networks in which 151 mouse genes were regulated (Supplementary Table 4). As shown in Figure 5, there were 32 genes (upregulated) that were detected in early and chronic stages of infection (i.e., highly targeted by *T. gondii*), and were involved in GO terms associated with biological processes relevant to the host-parasite interaction such as interspecies interaction between organisms; 34 genes (20 upregulated and 14 downregulated) exclusive to early infection were targeted by *T. gondii*, and the functional relevance of these interactions as shown by the enriched BPs were related to catabolism, biosynthesis, and metabolism-related processes; and 85 genes (64 upregulated and 21 downregulated) specific to chronic infection were more significantly enriched in specific molecular biosynthesis or localization, and metabolism process, such as small molecule biosynthetic process, macromolecule localization, and metabolic process.

## Discussion

In the present study, we compared the transcriptome of brain tissues of BALB/C mice infected by oocysts of the type II *T. gondii* Pru strain with that of uninfected mice at 11 and 33 dpi using RNA-seq profiling. The expression of 936 and 2,081 transcripts were altered during acute and chronic infection, respectively. This increased alterations of transcripts during chronic infection is in line with previous results obtained using tachyzoites of type II ME49 strain at acute (10 dpi) and chronic infection (28 dpi) (Pittman et al., 2014). A correlation between expressed host genes and developing cysts has been previously shown (Pittman et al., 2014; Garfoot et al., 2019a). Also, more enriched transcriptome profiles were found in the cysts (i.e., bradyzoite stage) compared to tachyzoite stage (Garfoot et al., 2019b). Long-term infection caused by *T. gondii* may produce greater changes of transcript abundance, reflecting more host response to the infection (Garfoot et al., 2019b).

In our study, we examined the transcriptomic changes for transcripts at two time points. As the infection progressed within the brain from early to late phases, the upregulated transcripts increased and so as the transcription of immune-related IFN-γ in response to combat the infection (Figure 3A). Also, functional enrichment analysis suggested that chronic infection leads to T cell activation and proliferation of immune cells, such as leukocytes and mononuclear cells (Figure 3A). IFN-γ, CD8^+^ T cells, *NF*-κ*B1* have been shown to mediate cell mediated immunity against *T. gondii* infection and contribute to the long-term resistance to *T. gondii* in the brain (Harris et al., 2010). Interestingly, the downregulation of mice response to calcium was significantly enriched in late infection (Supplementary Figure 1). In *T. gondii*, calcium-dependent protein kinases exhibited stage-specific expression, for example *CDPK5* was more abundant in bradyzoite stage (Pittman et al., 2014). Thus, it is possible that Ca^2+^ inside parasite cysts play a role in the regulation of host cell environment (Stewart et al., 2017; Zhang et al., 2018).

IFN-γ is a key cytokine in the control and elimination of *T. gondii* particularly in mice (Suzuki et al., 1988). IFN-γ killing of *T. gondii* is mediated by upregulation of the immunoregulatory enzyme indoleamine 2,3-dioxygenase (IDO) to deplete cellular tryptophan; inducible nitric oxide synthase (iNOS) to limit cellular arginine; IFN-γ inducible immunity-related GTPase (IRGs) and guanylate binding proteins (GBPs) to induce autophagy-dependent degradation of the parasitophorous vacuole membrane (PVM) (Collazo et al., 2001; Yarovinsky, 2014; Blader et al., 2015). In the present study, we revealed that IFN-γ (*Ifng*), iNOS (*Nos2*), six IRGs (*Irgm1*, *Irgm2*, *Igtp*, *Iigp1*, *Tgtp1*, and *Tgtp2*), and 11 GBPs (*Gbp2*, *Gbp2b*, *Gbp3*, *Gbp4*, *Gbp5*, *Gbp6*, *Gbp7*, *Gbp8*, *Gbp9*, *Gbp10*, and *Gbp11*) were commonly upregulated during acute and chronic infections (adjusted *P-*value < 0.05; | log2FC| > 1.5). Consistent with the previous finding reported in *T. gondii*-infected mouse liver (He et al., 2016a), many IRGs and GBPs-related genes were upregulated. Although increased expression of IDO in the brain as anti-toxoplasma mechanism to control the parasite replication has been reported in previous studies (Nagineni et al., 1996; Zhu et al., 2019), the expression level of IDO did not change in response to *T. gondii* infection in this study.

Inflammasome-dependent pyroptosis is an important host cell death pathway for controlling *T. gondii* (Blader et al., 2015; Zamboni and Lima-Junior, 2015). In the present study, we detected nine pyroptosis-related DETs in the infected brain, including three (*Casp4*, *Gsdmd*, and *Pycard*) that were upregulated in both early and late infections; five (*Casp1*, *Naip2*, *Naip5*, *Naip6*, and *Nlrp1b*) were upregulated and one (*Gsdme*) was downregulated in chronic infection. Among them, *Casp1* (Caspase-1, an inflammatory caspase molecule), *Pycard* (ASC, an adapter molecule), and *Nlrp1b* (a tandem *Nrlp1* paralog, a sensor molecule) are components of the canonical inflammasome (Zamboni and Lima-Junior, 2015). Consistent with this, inflammasome in response to *T. gondii* requires *Nrlp1b*-mediated *Casp1* activation during late infection (Ewald et al., 2014). Our results also showed that two pro-inflammatory cytokines, IL-1β and IL-18 related to *Casp1* activation (Rathinam et al., 2012), were upregulated during chronic infection (Supplementary Table 1), suggesting that these cytokines may help mice to mount an inflammatory response that prevents reactivation of latent *T. gondii* infection. In addition, the specificity of *Nrlp1b* activation depends on the polymorphism of the sensor molecule (Zamboni and Lima-Junior, 2015), whether this is a response specific to *T. gondii* or a generic response to any infection remains to be determined.

The present study detected 38 differentially expressed transcription factors (TFs) and 102 differentially expressed gene targets in *T. gondii*-infected brain tissues. There were 11 TFs commonly activated during early and late infections (Figure 4 and Supplementary Table 3). For example, the *Irf8* within the infected dendritic cells (DCs), especially the CD8^+^ DCs, has a critical role in detecting Toll-like receptor-mediated *T. gondii* profilin and in the subsequent induction of IL-12 by Myd88-mediated signaling pathway (Yarovinsky, 2014). The *Irf1* and *Stat1* contribute to the control of *T. gondii* by regulating the expression of factors essential for the host to resist infection, such as *Socs1* (Zimmermann et al., 2006), *Ciita* (Rosowski et al., 2014), and *NOS2* (Gavrilescu et al., 2004). We also detected 35 TFs exclusive to chronic infection that were involved in more interactions within the mouse brain (Table 2 and Figure 4 blue line color).

Previous data suggested correlated gene expressions between the host and the parasite, which can be attributed to the molecular interactions that mediate infection (Reid and Berriman, 2013). *T. gondii* can utilize its secretory effector proteins to manipulate the transcriptome and various signaling pathways in the host cells to its own benefits (Blader et al., 2015; Hakimi et al., 2017). To study host-pathogen interactomes, homology-based prediction is the one of most common approaches that has been applied to study a variety of intra-species interactions (Lee et al., 2008; Reid and Berriman, 2013; Cuesta-Astroz et al., 2019). More recently, bioinformatic analysis (Cuesta-Astroz et al., 2019) employed this strategy, and predicted effector molecules of *T. gondii* and their interactions with the host. There are certain ways by which *T. gondii* can evade host defense mechanisms, such as directly inactivating the IFN-γ-inducible GTPase by using effector molecules and avoiding IFN-dependent killing by targeting host signaling and gene transcription (Blader et al., 2015; Hakimi et al., 2017). By comparing the effect of *T. gondii*-related factors on mouse brain over the course of infection and distinguishing functional differences of the related DETs, we found that dysregulated transcripts expressed exclusively in early and late infection stages were significantly enriched in biochemical processes-related regulations (Figure 5). These results show the ability of *T. gondii* factors to alter the brain homeostasis, leading to neuropathology and disruption of neurotransmitter (David et al., 2016). Our analysis also revealed shared DETs between early and late infections which were significantly enriched in immune-related terms (Figure 5). Collectively, these results suggest that *T. gondii* has significant capabilities to manipulate host cell and can dysregulate many transcripts and processes in the mouse brain.

## Conclusion

RNA sequencing was used to characterize the transcriptional changes of the mouse brain during infection by *T. gondii* oocysts at 11 and 33 dpi. The differentially expressed transcripts (DETs) during late infection were nearly double the number of DETs during early infection. Most of the upregulated transcripts are shared between early and late infection stages, and the greatest difference detected in the downregulated transcripts. Acute infection-induced DETs were mainly associated with IFN-γ inducible immune response and host defense, and IFN-γ was also necessary to maintain chronic infection. Notably, chronic infection resulted in the activation of T-cells and the proliferation of other immune cells such as leukocytes and lymphocytes. *T. gondii* infection altered the expression of TFs and their target genes, particularly at late stage of infection. Significant interactions between *T. gondii* and the mouse brain DETs were also detected. These data improve our understanding of the transcriptomic changes that occur during *T. gondii* oocyst infection in mice and provide new insight into the neuropathogenesis of *T. gondii* infection.

## Data Availability Statement

The datasets supporting the findings of this article are included within the article and its Supplementary Material. The RNA-seq raw data described in the present study has been submitted to the NCBI Short Read Archive database (https://www.ncbi.nlm.nih.gov/sra) under the bio-project number PRJNA483261.

## Ethics Statement

The animal study was reviewed and approved by the experimental protocol of this study was reviewed and approved by Animal Research Ethics Committee of Lanzhou Veterinary Research Institute, Chinese Academy of Agricultural Sciences. The animals were handled in compliance with the animal ethics requirements of the People’s Republic of China. All efforts were made to minimize animal suffering during the experiment.

## Author Contributions

WC, J-JH, and X-QZ conceived and designed the study. R-SH, YZ, ME, Q-NM, and WC performed the experiments. R-SH, J-JH, HE, and WC contributed reagents, materials and analysis tools. R-SH and WC analyzed the data and wrote the manuscript. HE, J-JH, WC, and X-QZ critically revised the manuscript. All authors contributed to the article and approved the submitted version.

## Conflict of Interest

The authors declare that the research was conducted in the absence of any commercial or financial relationships that could be construed as a potential conflict of interest.

## Footnotes

1 https://rstudio.com/

## Supplementary Material

Supplementary Figure 1 (Top panel) the top 10 GO terms in the categories: biological process (BP), cellular component (CC) and molecular function (MF), and KEGG analysis of the enriched upregulated transcripts in the mouse brain during acute and chronic *T. gondii* infections. Bottom panel the top 10 GO terms of the enriched down-regulated transcripts. BP terms were not significantly enriched in acute infection, and CC and MF terms were not significantly enriched in chronic infection.

Supplementary Table 1 Differentially expressed transcripts identified in the present study during acute and chronic *T. gondii* infections.

Supplementary Table 2 Oligonucleotide primers and results of qRT-PCR analysis used to verify the sequencing data.

Supplementary Table 3 Details of the correlation between TFs and their target genes in the co-expression regulatory network.

Supplementary Table 4 Details of host-parasite interaction analysis, including *T. gondii*-related factors and their functions.
